# Targeting of ΔNp63α by miR‐522 promotes the migration of breast epithelial cells

**DOI:** 10.1002/2211-5463.13072

**Published:** 2021-01-10

**Authors:** Yuanyuan Dong, Juan Long, Xingyong Luo, Gang Xie, Zhi‐Xiong Jim Xiao, Ying Tong

**Affiliations:** ^1^ Center of Growth, Metabolism and Aging Key Laboratory of Bio‐Resource and Eco‐Environment of Ministry of Education College of Life Sciences Sichuan University Chengdu China; ^2^ Sichuan Integrative Medicine Hospital Chengdu China

**Keywords:** breast cancer, microRNA, migration, miR‐522, proliferation, ΔNp63α

## Abstract

The *TP63* gene, which encodes the p63 protein, is involved in multiple biological processes, including embryonic development and tumorigenesis. ΔNp63α, the predominant isoform of p63 in epithelial cells, acts as an oncogene in early‐stage tumors, but paradoxically acts as a potent antimetastatic factor in advanced cancers. Here, we report that ΔNp63α is a direct target of hsa‐miR‐522 (miR‐522). Induced expression of miR‐522 reduced the levels of ΔNp63α, predisposing breast epithelial cells to a loss of epithelial and acquisition of mesenchymal morphology, resulting in accelerated collective and single‐cell migration. Restoration of ΔNp63α repressed miR‐522‐induced migration. Interestingly, overexpression of miR‐522 did not affect breast epithelial cell proliferation, suggesting that miR‐522 acts specifically through ΔNp63α in this context. Furthermore, expression of miR‐522‐3p and p63 was negatively correlated in human cancer samples. Thus, miR‐522 might be a causative factor for breast tumorigenesis and cancer metastasis. In summary, our results reveal a novel miR‐522/p63 axis in cell migration and thus suggest a potential strategy for therapeutic treatment of cancer metastasis.

## Graphical abstract

p63 is a p53 family member with pivotal roles in tumorigenesis. Here, we report that hsa‐miR‐522 (miR‐522) is a direct regulator of p63. miR‐522 could promote scattering and migration of mammary epithelial cells through repressing ΔNp63α. The expression of miR‐522‐3p and p63 was negatively correlated in human cancers. The miR‐522/p63 axis might be a potential therapeutic target for cancer metastasis.

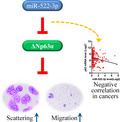

**Abbreviations**
EMT: epithelial–mesenchymal transition
F: forward
miR‐522: hsa‐miR‐522
R: reverse
SCC: squamous cell carcinoma
TCGA: The Cancer Genome Atlas
TGF‐β: transforming; growth factor‐β
TNBC: triple‐negative breast cancer
WT: wild type

The *TP63* gene, which encodes the p63 protein, is a member of the tumor suppressor *TP53* family with fundamental roles in multiple biological processes, including embryonic development, cell proliferation and tumorigenesis [1]. Alternative splicing of the *TP63* gene generates distinct protein isoforms, whereas ΔNp63α, which lacks the N‐terminal transactivation domain, is the predominant one in epithelial cells and epithelial‐originated tumors [2].

ΔNp63α may act as either a tumor promoter or a suppressor based on precise cellular scenarios. High expression of ΔNp63α is frequently observed in early‐stage cancers and function to stimulate tumorigenesis. It may enhance proliferation and survival of squamous cell carcinoma (SCC) through antagonizing p53/p73 signaling [3]. It can also cooperate with HRAS and AKT1 to drive tumorigenesis and induce chemoresistance on genotoxic stress [4, 5]. However, reduced p63 expression is often found in more progressive cancers with metastatic incidence and is linked to poor clinical outcomes [6, 7, 8, 9]. Further research reveals that the ΔNp63a isoform is the key player in tumor invasion and infiltration. It can reduce the MAPK1/MAPK2 activity to inhibit metastasis of breast cancer [6]. Many known metastatic suppressors, such as ID3 and CD82, can also be stimulated by ΔNp63α [10, 11]. In particular, ΔNp63a can target the miR‐205/ZEB1 axis to inhibit epithelial–mesenchymal transition (EMT) and metastasis of prostate and bladder cancers [7, 9]. In contrast, ΔNp63α overexpression promotes EMT in keratinocytes by regulating GRHL2, miR‐200 family genes and miR‐429 [12]. In osteosarcoma cells, ΔNp63α can repress miR‐527 and miR‐665 and aberrantly initiate a wound‐healing program to promote TGF‐β‐induced metastasis [13]. These conflicts might reflect the distinct roles associated with ΔNp63α in different cellular conditions.

In contrast, growing modulators have been identified that can regulate ΔNp63a to control the cell motility and cancer metastasis. Oncogenic PI3K, HRAS and ERBB2 have been reported to be able to suppress ΔNp63α expression to promote EMT and metastasis of lung and breast cancers [8]. TGF‐β and HRAS can cooperate with mutant‐p53 to induce metastasis of breast and squamous cancer cells by antagonizing p63 function [14]. Repression of ΔNp63α by SNAI1 also potentiates an invasive phenotype in SCC cells [15].

Among these factors, only a few microRNAs have been identified to be able to modulate the expression of p63 to regulate cellular migration. miR‐301a is able to directly inhibit p63 expression and induce EMT and invasion of prostate cancer cells [16]. In contrast, miR‐196a‐3p can suppress estrogen‐stimulated invasion of breast cancer cells through inhibition of p63 expression [17]. Interestingly, repression of p63 by miR‐223‐5p reduces migration but increases invasion of vulvar cancer cells [18], further revealing the complexity of the p63 regulatory network and putting forward the need for elaborate works to clarify the regulation of p63 on diverse cellular contextures.

hsa‐miR‐522 (miR‐522) is a member of the primate‐specific chromosome 19 microRNA cluster [19]. Accumulating evidences have shown that miR‐522 might exert oncogenic functions in the development of malignant tumors. miR‐522 is frequently found to be up‐regulated in different types of cancers, including hepatocellular carcinoma, non‐small‐cell lung cancer, colorectal cancer, glioblastoma and osteosarcoma [20, 21, 22, 23, 24, 25, 26, 27]. Overexpression of miR‐522 is considered an unfavorable prognostic biomarker [21, 27, 28, 29]. miR‐522 can enhance the proliferation, migration and invasion of non‐small‐cell lung cancer cells by interacting with DENND2D, SOCS5 and SFRP2 [23, 24, 30]. miR‐522 participates into the progression of hepatocellular carcinoma by activating Wnt signaling [20]. It can stimulate the TGF‐β signaling pathway to induce osteosarcoma tumorigenesis [31]. Binding to MTHFR empowers miR‐522 with the ability to enhance the risk for cervical cancer [32]. miR‐522 has also been reported to be able to boost colorectal tumorigenesis via targeting BLM [25], accelerate the progression of endometrial carcinoma by inhibiting MAOB [29], as well as promote glioblastoma cell proliferation by suppressing PHLPP1 [26]. Current studies even reveal that miR‐522 shuttled by exosomes can promote acquired chemoresistance in gastric cancer and non‐small‐cell lung cancer [33, 34]. Furthermore, miR‐522 can participate in the inflammation processes through interacting with PTGR1 and SOCS3 [35, 36]. Elevated levels of miR‐522 have been described in triple‐negative breast cancer (TNBC) cells. But interestingly, miR‐522 reduces cell proliferation but enhances migration of MDA‐MB‐468 TNBC cells [19]. These distinctive phenotypes indicate that the function of miR‐522 in breast cancer is more complicated and needs further investigation.

The Tet‐responsive expression system has been widely applied in biological studies to control the timing of gene expression *in vitro* and *in vivo*. The system includes Tet‐on and Tet‐off types, based on whether gene expression is allowed in the presence or absence of Tet or its derivative doxycycline [37]. The Tet‐inducible vectors have also been employed to drive the expression of microRNAs and proved to be effective tools for the study of the functions of microRNAs [38, 39].

Here, we report that miR‐522 is a regulator of ΔNp63α. The expression of ΔNp63α was directly repressed by miR‐522 in MCF10A, MCF7 and MDA‐MB‐231 breast epithelial cells. To examine the role of miR‐522 in breast tumorigenesis, we used a Tet‐on system to drive the inducible expression of miR‐522. The results showed that overexpression of miR‐522 led to morphological scattering and increased migration of breast epithelial cells, which could be interrupted by reintroduction of ΔNp63α. miR‐522 did not change the proliferation of these cells. Moreover, the expression of miR‐522‐3p was found to be inversely correlated with p63 RNA and protein levels in human cancers.

## Materials and methods

### Cell culture

Human mammary epithelial cells MCF10A were cultured in Dulbecco’s modified Eagle’s medium/F‐12 (1 : 1) (HYLSH3002301; Thermo Fisher Scientific, Shanghai, China) with 20 ng·mL^−1^ epidermal growth factor (PHG0311; Invitrogen, Shanghai, China), 100 ng·mL^−1^ cholera toxin (C8052; Sigma‐Aldrich, Shanghai, China), 10 µg·mL^−1^ insulin (CC101‐5MG; Macgene, Beijing, China), 500 ng·mL^−1^ hydrocortisone (H4001; Sigma‐Aldrich) and 5% of horse serum (Invitrogen). MCF7 breast cancer cells were cultured in minimum essential medium (SH30024.01; Thermo Fisher Scientific) supplemented with 10% FBS and 10 µg·mL^−1^ insulin. MDA‐MB‐231 breast cancer cells, HEK293 cells and HeLa cells were cultured in Dulbecco’s modified Eagle’s medium (HYLSH3002201; Thermo Fisher Scientific) with 10% FBS (SH30070.03; Thermo Fisher Scientific).

### Plasmids construction

MicroRNA screen vectors were generated by PCR amplification and subcloned into pLenti‐m3‐blasticidin vector. TP63‐3′ UTR sequence was amplified by genomic PCR using primer pair TP63‐3′ UTR‐forward (F) and TP63‐3′ UTR‐reverse (R), and then cloned into pmirGLO Dual‐Luciferase reporter to construct TP63‐3′ UTR‐wild type (WT) vector. Vectors with deletion of the predicted binding site of miR‐522‐3p in TP63‐3′ UTR, namely, TP63‐3′ UTR‐∆1‐4, were constructed using primer pairs: 522DL1‐F and 522DL1‐R, 522DL2‐F and 522DL2‐R, 522DL3‐F and 522DL3‐R, and 522DL4‐F and 522DL4‐R, respectively. To construct the vector with inducible expression of miR‐522, we amplified genomic DNA using primer 522TRE‐F and 522TRE‐R and cloned into pLVX‐TRE3G vector of Lenti‐X™ Tet‐On 3G Inducible Expression System (631187; Clontech, Beijing, China). All primers are listed in Table 1. ΔNp63α expression vector was kept in our laboratory [6].

**Table 1:** Primers used in this study.

Primer	Primer sequence (5ʹ–3ʹ)
TP63‐3′ UTR‐F	ACTGTCGACGCAAGTCTGAAAATCCCTGAGCA
TP63‐3′ UTR‐R	CAAGCGGCCGCGATGGAAGAATGGACAAATAGGCAA
522DL1‐F	AAATTGAGTTGCACTTATTGTTTAATTTACTTGTTTTGGA
522DL1‐R	CAAAACAAGTAAATTAAACAATAAGTGCAACTCAATTTTC
522DL2‐F	CACATCAAACCTTTGAGTAGTCCATTGCTTATTATGTAGG
522DL2‐R	ATAATAAGCAATGGACTACTCAAAGGTTTGATGTGGCA
522DL3‐F	CTGTCATTGCACATAAGCTTTAATTTTAAAGTGCAAAA
522DL3‐R	TTTTGCACTTTAAAATTAAAGCTTATGTGCAATGACAG
522DL4‐F	TTTTGTATTTTCATGAAAATTAGTAAGAATACCACATCAA
522DL4‐R	TTGATGTGGTATTCTTACTAATTTTCATGAAAATACAAAA
522TRE‐F	TTTGGATCCAGCTAACCTGCTGATTCTTTG
522TRE‐R	TAAGAATTCAGGTCGCAGTGAGCAGAGT

### Dual‐luciferase assay

HEK293 or HeLa cells were cotransfected with 400 ng microRNAs and 100 ng TP63‐3′ UTR‐WT, TP63‐3′ UTR‐∆1‐4 or pmirGLO vector alone. Cells were harvested 24 h later and lysed in Passive Lysis Buffer. Lysates were analyzed for firefly and renilla luciferase activities using the dual‐luciferase reagent assay system (E1960; Promega, Beijing, China) according to the manufacturer's instructions. Relative luciferase activity was calculated as the ratio of firefly/renilla activities and normalized to control.

### Viral infection

For viral harvest, 293T cells were transfected with pLVX‐Tet3G or pLVX‐TRE3G‐microRNA constructs along with psPAX2/pMD2.G packaging vectors by Lipofectamine 2000 (11668‐019; Life Technology, Shanghai, China). Forty‐eight hours later, the media were collected and filtered to remove debris. Viral particles were then concentrated by ultracentrifugation (20 000 r.p.m., 2 h at 4 °C). MCF10A, MCF7 or MDA‐MB‐231 cells were infected with pLVX‐Tet3G viral particles and selected with 2 mg·mL^−1^ neomycin to get stably infected cells. These cells were then infected with pLVX‐TRE3G‐microRNA viral particles and selected with 2 µg·mL^−1^ puromycin and 1 mg·mL^−1^ neomycin to establish the inducible cell lines.

### Western blot

Western protocols were described previously [40]. Antibodies include p63 (sc‐8431; Santa Cruz), β‐actin (M20010; Abmart, Shanghai, China), goat anti‐mouse IgG–horseradish peroxidase (sc‐2005; Santa Cruz Biotechnology, Shanghai, China) and goat anti‐rabbit IgG–horseradish peroxidase (sc‐2004; Santa Cruz Biotechnology).

### Wound‐healing assay

Cells were seeded into six‐well plates at a density of 1 × 10^6^ (MCF10A and MDA‐MB‐231) or 1.5 × 10^6^ (MCF7) cells per well. Twelve hours later, the monolayer of cells was wounded by performing a scratch with a sterile 200‐μL micropipette tip. The cells were washed with PBS and further cultured in media with 2% horse serum (MCF10A) or without serum (MCF7 and MDA‐MB‐231). Wound areas were monitored for 24 h (MCF10A and MDA‐MB‐231) or 72 h (MCF7).

### Transwell assay

Twenty‐four‐well transwell chambers (353097; BD Biosciences, Shanghai, China) were used according to the manufacturer's protocol. Cells (4 × 10^4^ for MCF10A, 2 × 10^5^ for MCF7 and 2.5 × 10^4^ for MDA‐MB‐231) were plated into the upper chamber with 200 µL serum‐free medium; 500 µL complete medium was added to the lower chamber as a chemoattractant. After incubation for 24 h (MCF10A and MDA‐MB‐231 cells) or 6 days (MCF7 cells), cells were fixed with methanol and stained with 0.1% crystal violet. Migrated cells in the lower chamber were imaged, and cell numbers (MCF10A and MDA‐MB‐231 cells) were counted by image j (National Institute of Mental Health, Bethesda, MD, USA) and normalized to control. Clones formed by migrated MCF7 cells were counted.

### Cell growth analysis

For growth analysis of MCF10A cells, 4 × 10^4^ cells were seeded into 12‐well plates and cultured under normal growth conditions. Every 24 h, cells were counted with a hemocytometer.

For growth analysis of MCF7 and MDA‐MB‐231 cells, 1 × 10^3^ cells per well were seeded into 96‐well plates, and cell viability assay (MTS, 3‐(4,5‐dimethylthiazol‐2‐yl)‐5‐(3‐carboxymethoxyphenyl)‐2‐(4‐sulfophenyl)‐2H‐tetrazolium, inner salt) was performed using the CellTiter 96 kit (G3582; Promega) according to the manufacturer's instructions.

### Bioinformatics analysis

All data were downloaded from the University of California Santa Cruz Xena portal [41] (https://xenabrowser.net/). Genomic Data Commons Data Portal The Cancer Genome Atlas (TCGA) Breast Cancer datasets were analyzed for RNA level of p63 or miR‐522. Datasets of lymph‐node‐negative breast cancer [42] were used to analyze the relapse profile. TCGA Breast Cancer and TCGA Pan‐Cancer datasets were analyzed for correlation between miR‐522‐3p mature RNA and p63 expression.

graphpad prism 6.0 (GraphPad Software Inc., San Diego, CA, USA) was used for data processing, calculation and graphing. Pearson's correlation coefficient was used to rank the correlation. Median expression value of miR‐522 or p63 was used as a cutoff to classify samples into high‐ and low‐expression groups. Student's *t* test was used for group comparisons.

## Results

### miR‐522 down‐regulates ΔNp63α expression via directly targeting its 3′ UTR

**Fig. 1:**
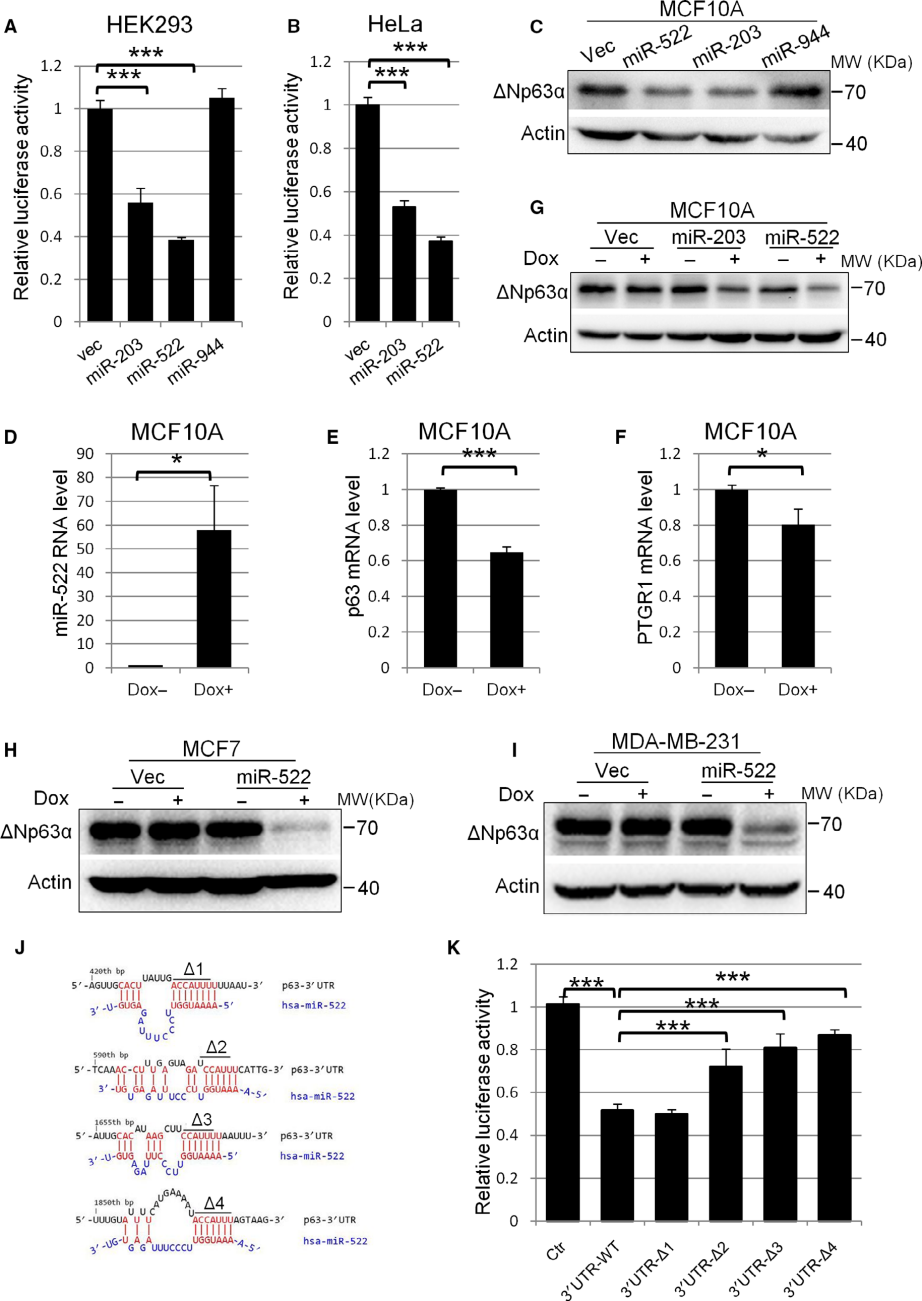
Ectopic expression of miR‐522 down‐regulates ΔNp63α expression via directly targeting its 3′ UTR. (A, B) Expression of miR‐522 significantly inhibits TP63 3′UTR‐luciferase activity. HEK293 (A) or HeLa (B) cells were cotransfected with TP63‐3′UTR‐WT and microRNA vectors or vector alone. Cell lysates were subjected to firefly and renilla luciferase activity assays. Relative luciferase activity was calculated as the ratio of firefly/renilla activities and normalized to control. (C) Expression of miR‐522 leads to down‐regulation of endogenous ΔNp63α in MCF10A cells. MCF10A cells were transiently transfected with miR‐522 vector or vector alone, and cell lysates were subjected to western blot. (D) Induced expression of miR‐522 on doxycycline treatment. Cells stably infected with inducible miR‐522 vectors were treated with or without doxycycline. Quantitative PCR was performed to check the RNA level of miR‐522. (E, F) Induced expression of miR‐522 leads to down‐regulation of ΔNp63α (E) and PTGR1 (F) mRNA levels in MCF10A cells. Cells were treated as in (D). (G–I) Induced expression of miR‐522 leads to down‐regulation of ΔNp63α protein level in MCF10A (G), MCF7 (H) and MDA‐MB‐231 (I) cells. Cells were treated as in (D), lysed and subjected to western blot. (J) Predicted miR‐522‐3p binding sequences on 3′UTR of TP63. Deleted sequence in the seed regions (∆1–4) is highlighted. (K) Deletions of the seed regions (Δ2–4) in the miR‐522‐3p binding sites interfere with miR‐522's effects on TP63. HEK293 cells were cotransfected with miR‐522 and TP63‐3′ UTR‐WT, TP63‐3′ UTR‐∆1‐4 or pmirGLO vector alone. Cell lysates were subjected to luciferase activity assays. All data are presented as means ± standard error of the mean. *n* = 3. *P < 0.05, ***P < 0.001, *t*‐test.

We first applied online bioinformatic algorithms [43, 44] to screen for potential microRNAs that can target TP63 3′ UTR. Candidate microRNAs were cloned into lentiviral vector pLenti‐m3 and cotransfected with dual‐luciferase vectors harboring TP63 3′ UTR (TP63‐3′ UTR‐WT) into HEK293 or Hela cells. miR‐203, a well‐defined regulator of p63, was used as the positive control. Among the predicted microRNAs, transfection of miR‐522 significantly reduced the luciferase activity to 38.5% of the control level in HEK293 cells and 37.1% in HeLa cells (*P* < 0.001), suggesting a putative inhibition of p63 expression by miR‐522. miR‐203 decreased the reporter activity to a similar extent, whereas miR‐944 showed no significant effects (Fig. 1A,B).

We overexpressed miR‐522 in mammary epithelial MCF10A cells, in which ΔNp63α is the predominant p63 isoform. Western blot showed that miR‐522 overexpression reduced the endogenous ΔNp63α level, comparable with miR203 control (Fig. 1C). We then used the Tet‐On 3G inducible expression system to establish stable MCF10A cell lines with inducible expression of miR‐522. Quantitative PCR confirmed that treatment of these cells with 1 µg·mL^−1^ doxycycline for 48 h caused expression of miR‐522, accompanied with down‐regulation of p63 and PTGR1 (another miR‐522 target verified in inflammation processes) (Fig. 1D–F). Western blot further showed that induced expression of miR‐522 led to distinct decrease of the ΔNp63 protein level. These results indicated that miR‐522 could inhibit the expression of ΔNp63α through promoting its RNA degradation (Fig. 1G). Furthermore, in epithelial‐originated breast cancer MCF7 and MDA‐MB‐231 cells, induced expression of miR‐522 also led to a dramatic decrease of ΔNp63α expression (Fig. 1H,I). These results suggested that ΔNp63α was a functional target of miR‐522.

A bioinformatics algorithm found four potential miR‐522‐3p binding sites at the 3′UTR of TP63 (Fig. 1J). To check whether miR‐522‐3p specifically inhibits p63 by binding to these sites, we constructed reporter vectors with deletion on the seed sequence of these sites, namely, TP63‐3′ UTR‐∆1‐4. TP63‐3′ UTR‐WT or TP63‐3′ UTR‐∆1–4 were cotransfected with miR‐522 into HEK293 cells, and luciferase activities were analyzed. Compared with the control vector, miR‐522 significantly reduced the reporter luciferase activity when cotransfected with TP63‐3′ UTR‐WT. Deletion of ∆1 sequence did not interfere with this function. However, deletion of ∆2–4 sequences increased the luciferase activity to 72.1%, 80.8% and 86.8%, respectively (*P* < 0.001), indicating that these three sites were critical for miR‐522‐3p to bind to TP63 3′ UTR (Fig. 1K). These results demonstrated a direct targeting of TP63 3′ UTR by miR‐522‐3p.

### miR‐522 induces mesenchymal‐like morphology of breast epithelial cells, which can be repressed by overexpression of ΔNp63α

**Fig. 2:**
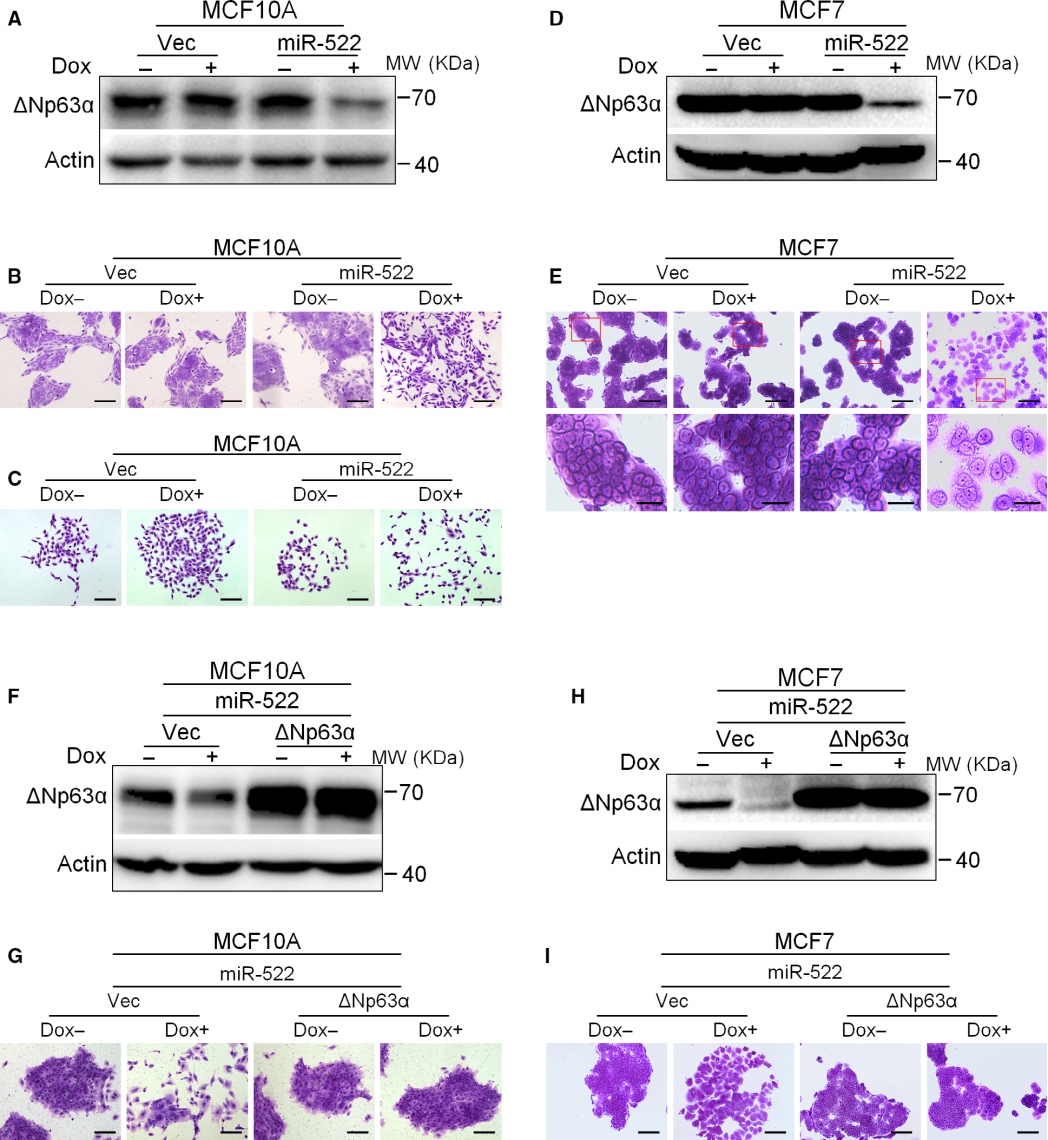
miR‐522 induces mesenchymal‐like morphology of breast epithelial cells, which can be repressed by overexpression of ΔNp63α. (A) miR‐522 leads to down‐regulation of ΔNp63α in MCF10A cells. Stable cells with inducible miR‐522 or control vectors were treated with or without doxycycline and subjected to western blot. (B, C) miR‐522 induces the mesenchymal‐like morphology of MCF10A cells. Cells were treated as in (A) and reseeded into six‐well plate (B), or single cells were reseeded to get cell clones (C). Scale bars: 200 µm. (D) miR‐522 leads to down‐regulation of ΔNp63α in MCF7 cells. Cells were treated as in (A) and subjected to western blot. (E) miR‐522 induces the mesenchymal‐like morphology of MCF7 cells. Cells were treated as in (A) and reseeded into six‐well plate. Boxed areas in the pictures were enlarged below. Scale bars: 100 µm for original images; 30 µm for enlarged images. (F) Overexpression of ΔNp63α in MCF10A cells with inducible miR‐522 vector. Cells were stably infected with pLVX‐ΔNp63α or vector alone, treated with or without doxycycline and subjected to western blot. (G) ΔNp63α represses the morphological changes of MCF10A cells induced by miR‐522. MCF10A cells were treated as in (F) and reseeded into a six‐well plate. Scale bars: 200 µm. (H) Overexpression of ΔNp63α in MCF7 cells with inducible miR‐522 vector. Cells were treated as in (F) and subjected to western blot. (I) ΔNp63α represses the morphological changes of MCF7 cells induced by miR‐522. Cells were treated as in (F) and reseeded into a six‐well plate. Scale bars: 100 µm.

Cultured MCF10A epithelial cells associated tightly with cell–cell junctions. When miR‐522 was induced to be expressed in them, the cells became dissociated and shifted toward mesenchymal‐like morphology (Fig. 2A,B). Meanwhile, the single‐cell clones became more scattered compared with the control groups (Fig. 2C). In tightly associated MCF7 cells, induced expression of miR‐522 also stimulated the dissociation and scattering of cells (Fig. 2D,E).

We then investigated the function of p63 in 522‐induced scattering phenotype. The results indicated that ectopic expression of ΔNp63α in MCF10A and MCF7 cells could repress the scattering response and make cells associated tightly again (Fig. 2F–I). Thus, ΔNp63α might participate in the epithelial scattering pathway of miR‐522.

EMT is the pivotal step in cancer metastasis; our results suggested that the miR‐522/p63 axis might play important roles in epithelial cell scattering and thus contribute to cell migration.

### miR‐522 promotes the migration of breast epithelial cells, which can be repressed by overexpression of ΔNp63α

**Fig. 3:**
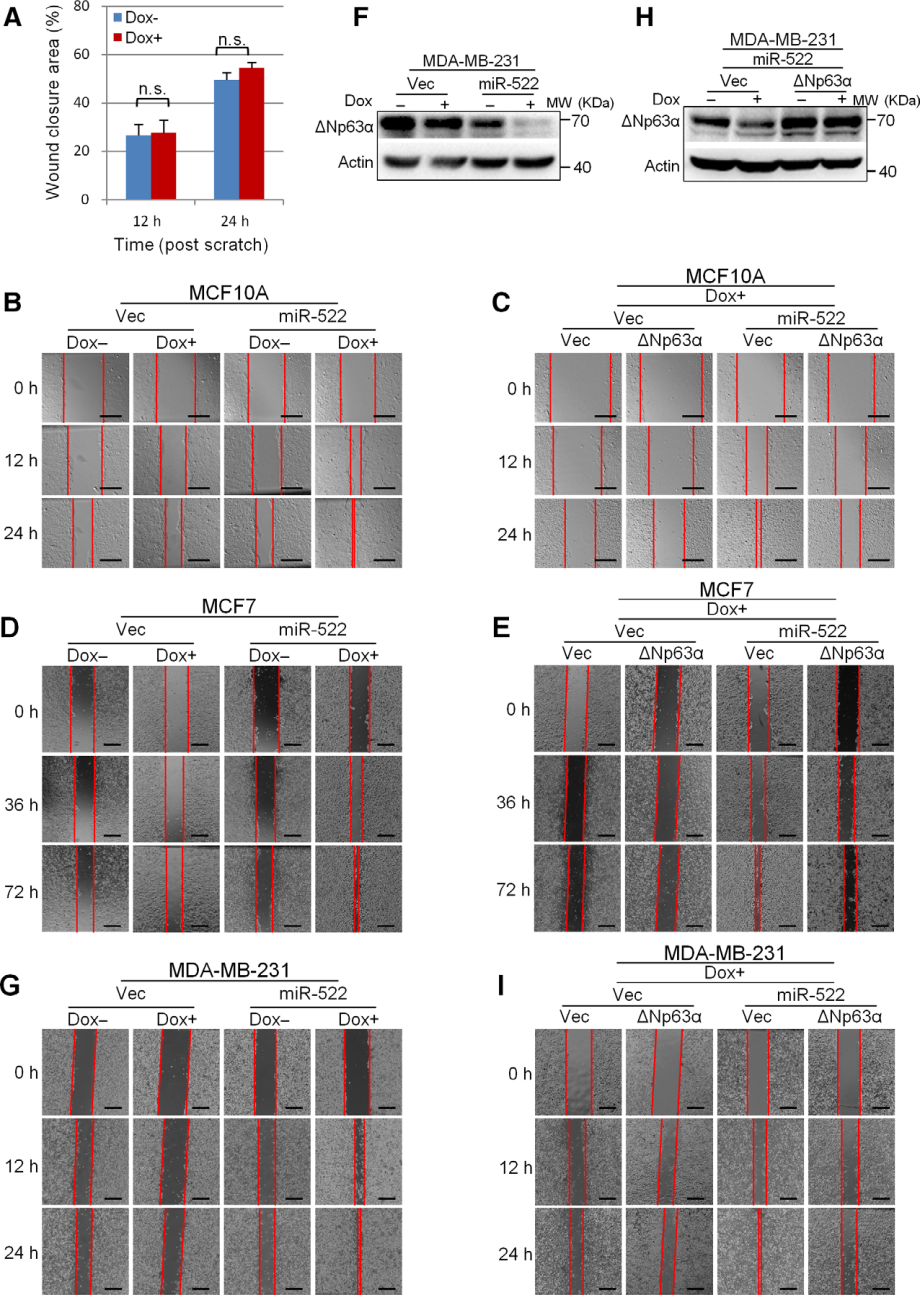
miR‐522 promotes the collective migration of breast epithelial cells, which can be repressed by overexpression of ΔNp63α. (A) Doxycycline treatment does not significantly change the wound‐healing speed. MCF10A cells were treated with or without doxycycline and reseeded into a six‐well plate. Wound healing was evaluated by performing scratching. Wound closure areas were calculated and normalized to areas at 0 h. Data are presented as means ± standard error of the mean. *n* = 3. n.s., nonsignificant, *t*‐test. (B) miR‐522 promotes the collective migration of MCF10A cells. Stable MCF10A cells harboring miR‐522 vector or control were treated as in (A). (C) ΔNp63α inhibits the migration of MCF10A cells induced by miR‐522. MCF10A cells were stably infected with pLVX‐ΔNp63α or vector alone. Cells were treated with doxycycline and then subjected to wound‐healing assays as in (A). (D) miR‐522 promotes the collective migration of MCF7 cells. Cells were treated as in (A). (E) ΔNp63α inhibits the migration of MCF7 cells induced by miR‐522. Cells were treated as in (C). (F) miR‐522 leads to down‐regulation of ΔNp63α in MDA‐MB‐231 cells. Cells were treated as in Fig. 2A and subjected to western blot. (G) miR‐522 promotes the collective migration of MDA‐MB‐231 cells. Cells were treated as in (A). (H) Overexpression of ΔNp63α in MDA‐MB‐231 cells with inducible miR‐522 vector. Cells were treated as in Fig. 2F and subjected to western blot. (I) ΔNp63α inhibits the migration of MDA‐MB‐231 cells induced by miR‐522. Cells were treated as in (C). Scale bars: 300 µm (B, C); 600 µm (D, E, G, I).

To further investigate the function of miR‐522 in cell migration, we performed wound‐healing assays in MCF10A, MCF7 and MDA‐MB‐231cells cultured with reduced serum concentration. We confirmed that treatment with doxycycline did not significantly affect the wound closure speed in control MCF10A cells (Fig. 3A). However, in cells harboring inducible miR‐522 vector, doxycycline treatment turned on the ectopic expression of miR‐522 and markedly increased the wound closure speed, indicating that miR‐522 can promote the collective migration of MCF10A cells (Fig. 3B). In addition, overexpression of ΔNp63α could counteract with miR‐522's function and slow down the wound closure (Fig. 3C). Wound‐healing assays were then performed in MCF7 and MDA‐MB‐231 cells. The results further demonstrated that miR‐522 could accelerate the wound closure speed and ΔNp63α could intervene in this process (Fig. 3D–I).

**Fig. 4:**
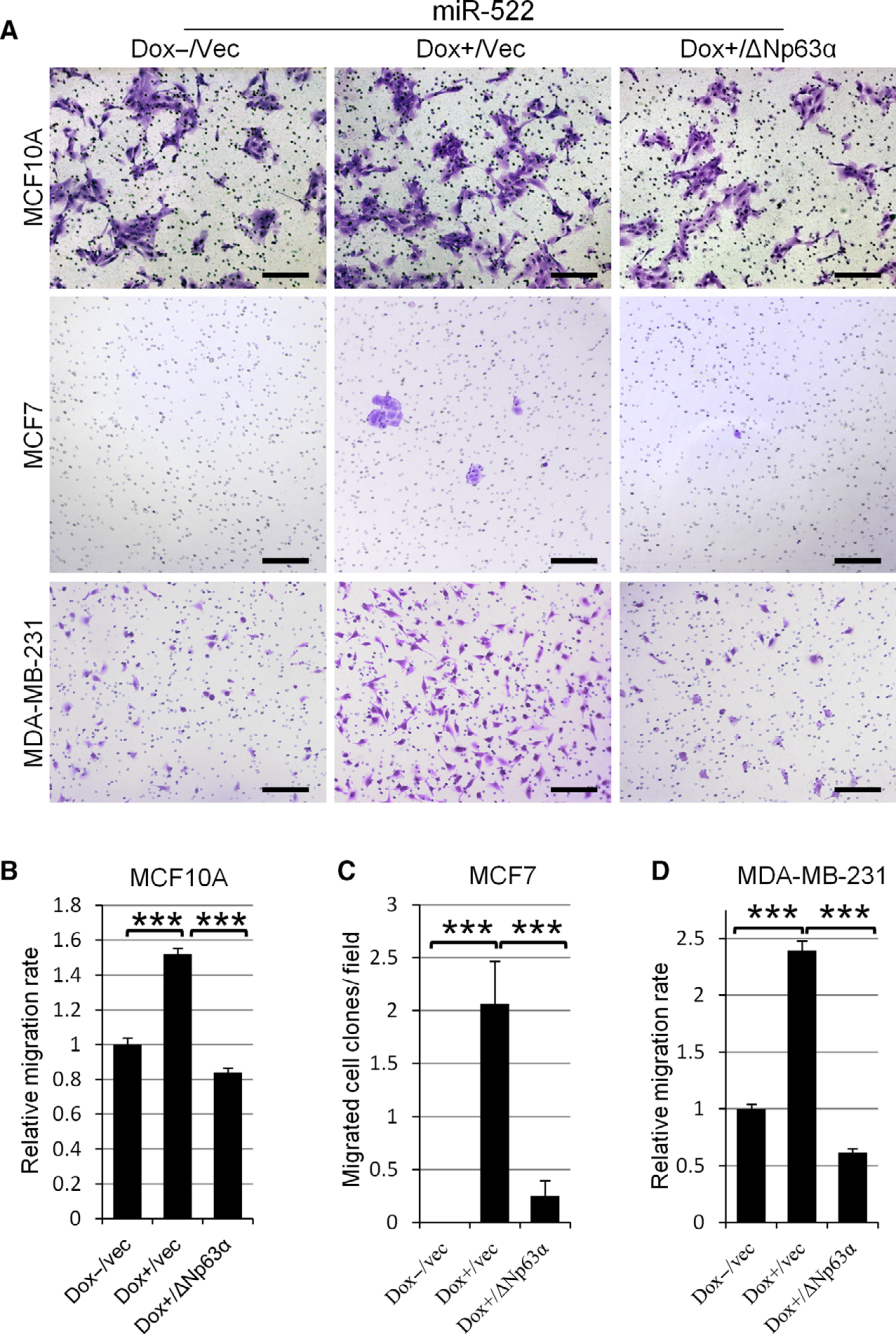
miR‐522 promotes the single‐cell migration of breast epithelial cells, which can be repressed by overexpression of ΔNp63α. (A) miR‐522 promotes the single‐cell migration of breast epithelial cells, and ΔNp63α inhibits its effects. MCF10A, MCF7 and MDA‐MB‐231 cells with inducible miR‐522 vector were stably infected with pLVX‐ΔNp63α or vector alone. Cells were treated with or without doxycycline and then subjected to transwell assays. Migrated cells were imaged. Scale bars: 200 µm. (B–D) Transwell assays were evaluated. The numbers of migrated cells were counted and normalized to control (B, D). The numbers of migrated cell clones were counted (C). Data are presented as means ± standard error of the mean. *n* = 3. ***P < 0.001, *t*‐test.

We then conducted transwell assays to check whether miR‐522 can modulate the single‐cell migration. When miR‐522 was induced to be expressed by doxycycline treatment, more MCF10A cells migrated through the transwell and showed 52.0% higher migration rate. Infection of ΔNp63α into these cells led to a dramatic reduction of the migration rate, indicating that ΔNp63α could abolish miR‐522's migration‐promoting effect (Fig. 4A,B).

In transwell assays, MCF7 cells associated so tightly that they could not pass the membrane. Thus, we cultured the cells for 6 days and counted the cell clones formed by migrated cells. The results showed that induced expression of miR‐522 empowered MCF7 cells with the ability to pass through the membrane, and ΔNp63α could reverse this effect (Fig. 4A,C). Similar results were recapitulated in MDA‐MB‐231 cells (Fig. 4A,D).

**Fig. 5:**
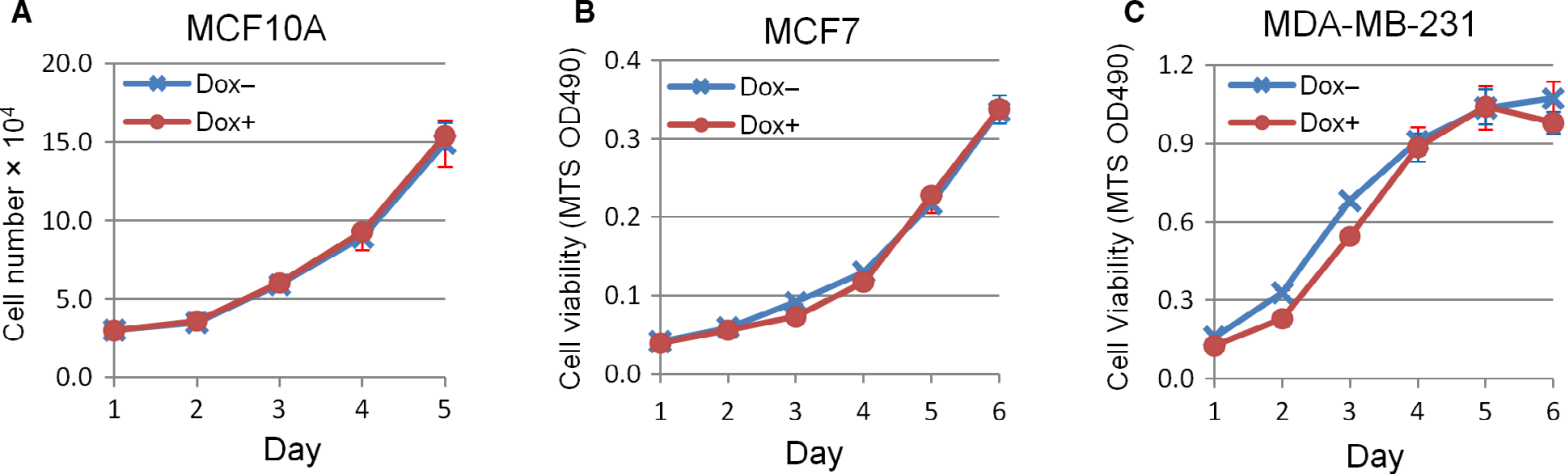
miR‐522 does not affect the proliferation of breast epithelial cells. (A) miR‐522 does not affect the proliferation of MCF10A cells. MCF10A cells with inducible miR‐522 vector were cultured in media with or without doxycycline. Cell numbers were counted for 5 consecutive days at 24‐h intervals. Data are presented as means ± standard error of the mean (SEM). *n* = 3. (B, C) miR‐522 does not affect the viability of MCF7 (B) and MDA‐MB‐231 (C) cells. Cells were treated as in (A) and subjected to MTS analysis. Data are presented as means ± SEM. *n* = 2.

Further experiments demonstrated that doxycycline treatment did not significantly change the growth rate of cultured MCF10A, MCF7 and MDA‐MB‐231 cells harboring inducible miR‐522 vector (Fig. 5A–C). Taken together, these results revealed that the miR‐522/p63 axis could regulate the migration of breast epithelial cells, whereas it did not affect cell proliferation.

### miR‐522‐3p inversely correlates with p63 expression in human cancer

**Fig. 6:**
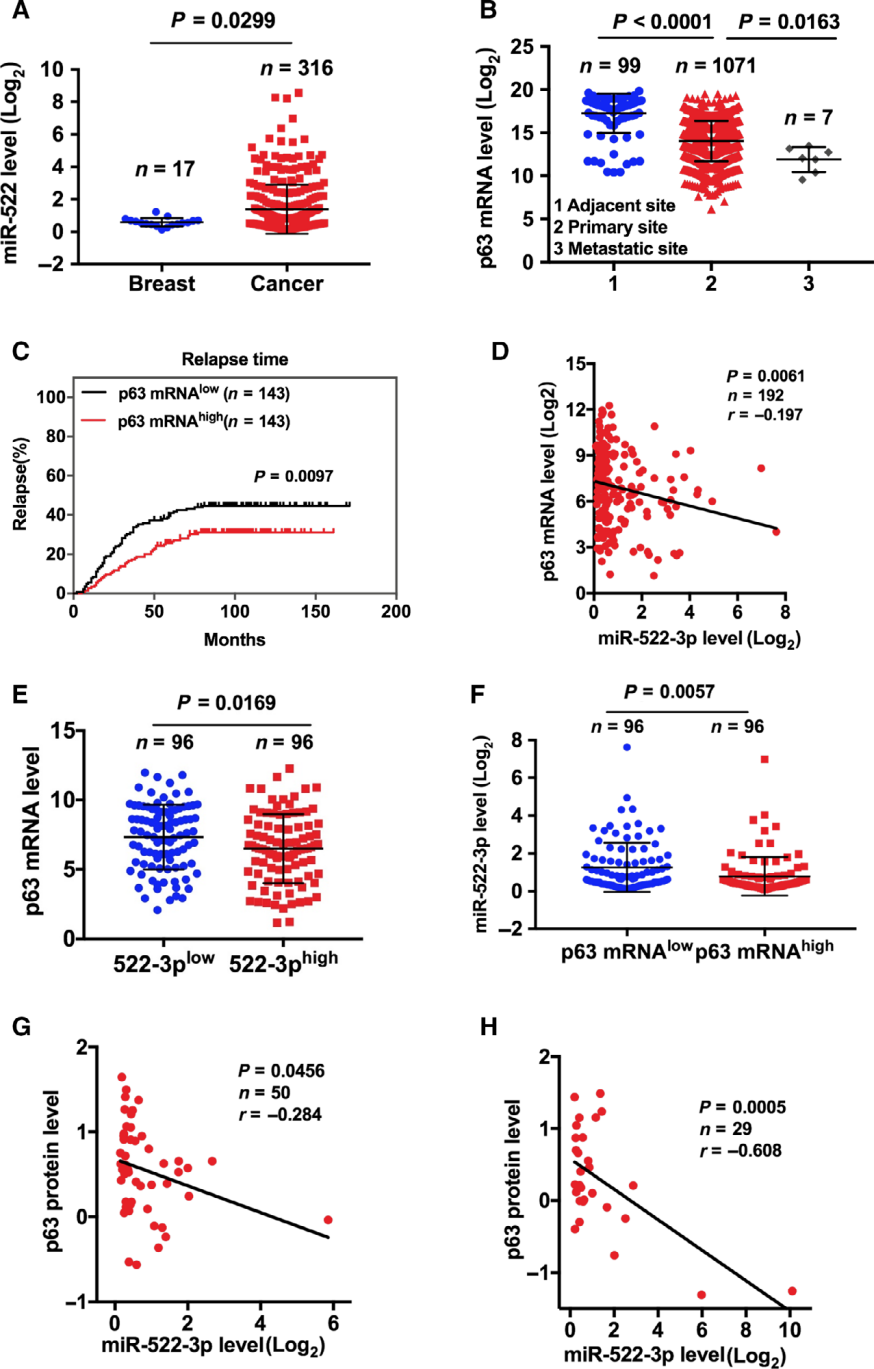
Correlation of miR‐522‐3p and p63 expression in human cancers. (A, B) Datasets of Genomic Data Commons Data Portal TCGA breast invasive cancer were analyzed for miR‐522 level (A) or p63 mRNA level (B). (C) Relapse profile of lymph‐node‐negative primary breast cancers. (D–F) Datasets of TCGA breast invasive cancer were analyzed for correlation of miR‐522‐3p and p63 RNA level. (G, H) Datasets of TCGA head and neck SCC (G) and lung SCC (H) were analyzed for correlation between miR‐522‐3p and p63 protein levels.

To further define the function of miR‐522 on p63, we explored bioinformatics databases for the clinical relevance of miR‐522 and p63 expression. Significant up‐regulation of miR‐522 was found in TCGA breast invasive carcinoma when compared with normal breast tissue (Fig. 6A). On the contrary, the expression of p63 showed a marked decrease in TCGA breast invasive carcinoma, especially in metastatic samples (Fig. 6B). In Wang's [42] study focusing on the metastasis of lymph‐node‐negative primary breast cancer, we found that patients with a high level of p63 showed a longer relapse time, further indicating the importance of p63 in regulating the metastasis of breast cancer (Fig. 6C).

In TCGA breast invasive carcinoma samples, Pearson’s analyses revealed a clear negative correlation between miR‐522‐3p mature RNA and p63 mRNA levels (Fig. 6D). We then classified samples into high‐ and low‐expression groups using the median value as a cutoff point. Statistical analysis revealed that samples with higher miR‐522‐3p expression demonstrated a significantly lower p63 level and vice versa (Fig. 6E,F). Notably, in 50 head and neck SCC samples with data for both protein level of p63 and miR‐522‐3p mature RNA expression, we found a significant inverse correlation (Fig. 6G). Moreover, p63 protein expression again showed a strong negative correlation with miR‐522‐3p mature RNA level in TCGA lung SCCs (Fig. 6H). Taken together, these data indicated that miR‐522‐3p inversely correlated with p63 expression in human cancers.

## Discussion

Here, we identified miR‐522 as a direct upstream regulator of p63. By targeting ΔNp63a, miR‐522 could promote migration of mammary epithelial cells while not affecting their proliferation. Furthermore, the expression of miR‐522‐3p demonstrated an inverse correlation with p63 level in human cancers.

In Tan et al.'s [19] work, pulldown sequencing isolated bunches of genes that could be pulled down by miR‐522 mimics in MDA‐MB‐468 TNBC cells. However, p63 was not identified as a target of miR‐522 in this work. Our bioinformatics and functional screen confirmed the direct regulation of p63 by miR‐522. Thus, it will be worthwhile to identify functional targets of microRNAs through alternate approaches.

Several experimentally validated targets of miR‐522 in human cancers have been documented, including ABCB5, BLM, MAOB, PHLPP1, DKK1, SFRP2, DENND2D, SOCS5 and PPM1A [20, 23, 24, 25, 26, 29, 30, 31, 45]. PTGR1 and SOCS3 are reported to be regulated by miR‐522 to participate into the inflammation processes [35, 36]. FOXA1, E2F3 and TFDP1 are also identified as miR‐522 targets by pulldown sequencing in breast cancer cells [19]. We have checked the mRNA level of these genes in TCGA breast invasive carcinoma and found that FOXA1, PTGR1 and MTHFR showed an obvious negative correlation with miR‐522 level. We then examined the expression of these three genes in MCF10A cells by quantitative PCR and found that PTGR1 could be repressed by miR‐522. It will be interesting to further screen for functional targets of miR‐522 and investigate their roles in breast cancer.

miR‐522 has been reported as a migration‐promoting factor for cancer cells. Overexpression of miR‐522 led to enhanced expression of EMT marker genes and invasive ability in MDA‐MB‐468 breast cancer cells [19]. In keeping with this experiment, we found that induced expression of miR‐522 in MCF10A and MCF7 breast epithelial cells promoted a shift toward mesenchymal morphology and an increase in cell migration. These results suggested that miR‐522 might be a causative factor in breast tumorigenesis through empowering breast epithelial cells with the migration potential and thus seeding the malignant risk from the initial stage.

miR‐522 is frequently considered as an oncogene, yet distinct results have been observed regarding its role in cell proliferation. Overexpression of miR‐522 could promote proliferation of hepatocellular carcinoma through repressing DKK1 and SFRP2 [20]. By targeting BLM, DENND2D, PHLPP1 or MAOB, miR‐522 could boost tumorigenesis of colorectal cancer, non‐small‐cell lung cancer, glioblastoma and endometrial carcinoma, respectively [20, 25, 26, 29]. Furthermore, miR‐522 could facilitate osteosarcoma tumorigenesis through regulating PPM1A expression [31]. However, overexpression of miR‐522 in MDA‐MB‐468 TNBC cells caused G1/S arrest and reduced cell proliferation [19]. Remarkably, miR‐522 could also sensitize colon cancer cells to doxorubicin treatment by targeting ABCB5, indicating its proliferation‐inhibiting property under specific circumstances [45].

In our experiments, we did not detect a significant change of cell proliferation on miR‐522 overexpression in MCF10A, MCF7 and MDA‐MB‐231 cells. This discrepancy might reflect the unique profile of the miR‐522/p63 axis in mammary epithelial cells. ΔNp63α may act either as a tumor promoter or a suppressor based on precise cellular scenarios. It may initiate tumorigenesis via promoting cell proliferation but inhibit tumor metastasis and progress in advanced cancers [3, 4, 5, 6, 7, 8, 9, 10, 11]. In our study, inhibition of ΔNp63α by miR‐522 might repress its antimigration property while concomitantly restraining its proliferation‐provoking ability, resulting in enhanced migration without increased proliferation.

Taken together, this study is the first to show that miR‐522 plays an important role in cell migration through repressing the expression of ΔNp63α. Our finding provides clues for the functions of the miR‐522/ΔNp63α axis in tumor metastasis. Molecular drugs targeting miR‐522 could be evaluated for cancer treatment, especially for metastatic tumors with reduced ΔNp63α level.

## Conflict of interest

The authors declare no conflict of interest.

## Author contributions

YT conceived and designed the project. YD, JL and XL performed experiments and data analysis. YD and YT wrote the manuscript. GX, Z‐XJX and YT contributed to the discussion and manuscript revision.

## Data Availability

The data that support the findings of this study are available from the corresponding author upon reasonable request.
